# FBW7 Regulates the Autophagy Signal in Mesangial Cells Induced by High Glucose

**DOI:** 10.1155/2019/6061594

**Published:** 2019-04-21

**Authors:** Chenlin Gao, Fang Fan, Jiao Chen, Yang Long, Shi Tang, Chunxia Jiang, Yong Xu

**Affiliations:** ^1^Department of Endocrinology, Affiliated Hospital of Southwest Medical University, Sichuan 646000, China; ^2^Faculty of Chinese Medicine, Macau University of Science and Technology, Avenida Wai Long, Taipa, Macau; ^3^Department of Endocrinology, The First People's Hospital of Neijiang, Sichuan 641000, China; ^4^Department of Endocrinology, The Third Hospital of Mianyang, Sichuan 621000, China

## Abstract

**Aims:**

Abnormal regulation of autophagy participates in the development of diabetic nephropathy. mTOR is the most common negative regulator of the autophagy signaling pathway. FBW7 constitutes the SCF (Skp1–Cullin1–F-box protein) recognition subunit of E3 ubiquitin ligase, and mTOR is a substrate of FBW7 that can be modified by ubiquitination and be degraded via proteasomes. In this study, we explored the relationship between FBW7 and autophagy and examined the effects of FBW7 on the occurrence of diabetic nephropathy in vitro.

**Materials and Methods:**

We cultured mesangial cells induced by high glucose in vitro and used rapamycin as a specific mTOR inhibitor, performed FBW7 gene overexpression, and detected the expression of autophagy signal and inflammatory factors by WB, ELISA, RT-PCR, and immunofluorescence.

**Results:**

High glucose can downregulate the expression of FBW7 and activate mTOR signal, which leads to diminished autophagy in renal mesangial cells, as well as renal inflammatory cytokines and fibrotic factors. RAPA, as a specifically inhibitor of mTOR, can decrease inflammatory cytokines and fibrotic factors by inhibiting mTOR. Moreover, FBW7 gene overexpression can increase autophagy by inhibiting mTOR signal; at the same time, the inflammatory cytokines and fibrotic factors were decreased in mesangial cells.

**Conclusions:**

FBW7 was decreased in renal mesangial cells induced by high glucose, and FBW7 gene overexpression can increase autophagy by inhibiting mTOR signaling and ameliorate inflammation and fibrosis.

## 1. Introduction

The worldwide prevalence of diabetes mellitus (DM) is increasing rapidly. Approximately 20% to 40% of patients with DM go on to experience diabetic nephropathy (DN), a primary cause of chronic kidney disease that necessitates dialysis or renal transplantation for survival in the end stage [1]. In the early stage, DN can be prevented or delayed by strictly controlling blood glucose. The mechanism of DN induced by hyperglycemia is unclear, but inflammation and fibrosis are known to be important pathophysiological processes in DN. Moreover, type I collagen (Col-I) is an established marker of fibrosis, and interleukin-1*β* (IL-1*β*) and cysteinyl aspartate specific protease-1 (caspase-1) are inflammatory markers. Studies have shown that autophagy is the central link in the development of DN [2].

Autophagy is a “self-feeding” phenomenon in cells. Autophagic vesicles coat proteins or organelles and then fuse with lysosomes to form autolysosomes. The enzymes of an autolysosome can degrade senescent organelles, redundant proteins, and harmful cytoplasmic components [3]. Autophagy can provide raw materials for organelle renewal and for cell repair, reconstruction, and stability.

Autophagy is not only a cellular “garbage-disposal plant” but also is a “waste recycle bin.” By means of autophagy, the cell can resist the invasion of pathogens and can be protected from damage by intracellular toxins. So, autophagy is an important defense and stress-regulation mechanism. Recent studies demonstrate that autophagy plays an important role in inflammatory diseases, chronic liver fibrosis, DM, and so on [4]. Some researchers found that autophagy can suppress inflammation and fibrosis to protect the kidney [5, 6]; dysregulation of autophagy can lead to the development of DN [7]. Moreover, the level of autophagy was lower in DN mouse than in normal mice [8]. P13K-Akt-mTOR constitutes the most common negative-regulatory signaling pathway of the cell; mTOR, the substrate of FBW7, can be modified by ubiquitination and degraded by proteasomes [9].

The ubiquitin proteasome system is complex and can degrade proteins selectively. Substrate selection is controlled by E3 ubiquitin ligase. FBW7 is the substrate recognition component of the Skp1–Cullin1–F-box (SCF) ubiquitin ligase complex [10]. Expression of FBW7 is associated with degradation of substrate proteins because FBW7 links ubiquitin to the protein [11]. In renal cell carcinoma, FBW7 negatively regulates the mTOR signaling pathway [12]. Increased expression of FBW7 promotes the degradation of mTOR and inhibits the mTOR signaling pathway; the result is to elevate autophagy levels to combat prion infection [13]. However, the expressions of mTOR signaling molecules have not been characterized in DN, and it is unknown whether FBW7 is involved in the regulation of the mTOR signaling pathway in the pathogenesis of DN. We cultured mesangial cells and determined FBW7 expression under high-glucose conditions to evaluate the regulation of autophagy and provide a novel theoretical basis for DN.

## 2. Materials and Methods

### 2.1. Cell Culture

SV40 MES 13 cells were purchased from the Chinese Academy of Sciences and were cultured in low-glucose Dulbecco's Modified Eagle's Medium (DMEM, HyClone, USA). To test the expression levels of FBW7 in renal mesangial cells induced by different concentrations of glucose, cells were divided into 5 groups: the normal control group (NC, 5.6 mmol/L of glucose), the osmotic pressure control group (OP, 5.6 mmol/L of glucose and 24.4 mmol/L of mannitol), the high-glucose group 1 (HG1, 10 mmol/L of glucose), the high-glucose group 2 (HG2, 20 mmol/L of glucose), and the high-glucose group 3 (HG3, 30 mmol/L of glucose).

To determine whether mTOR protein is involved in the regulation of autophagy, inflammation, and fibrosis, rapamycin (RAPA; Solarbio, China) was used as a specific inhibitor of mTOR, and renal mesangial cells were induced by 30mmol/L glucose for 48h and they were divided into 4 groups. Cells in the normal control (NC) group were maintained in 5.6 mmol/L of glucose; cells in the high-glucose group 3 (HG3) were maintained in 30 mmol/L of glucose; cells in the NC-RAPA3 group were cultured in 5.6 mmol/L of glucose and 200 nmol/L of rapamycin; cells in the HG3-RAPA3 group were treated with 30 mmol/L of glucose and 200 nmol/L of rapamycin.

To determine whether decreased autophagic activity is caused by inhibition of FBW7, we induced overexpression of FBW7 (Gene, China). Toward this end, cells were divided into 4 groups. Cells in the NC-LV-CON238 group were transfected with negative-control virus (CON238) and were cultured with 5.6 mmol/L of glucose for 48 hours. Cells in the NC-LV-FBW7 group were transfected with a lentiviral construct that overexpressed FBW7 and then were cultured with 5.6 mmol/L of glucose for 48 hours. Cells in the HG3-LV-CON238 group were transfected with negative-control virus (CON238) and were cultured with 30 mmol/L of glucose for 48 hours. Cells in the HG3-LV-FBW7 group were transfected with a lentiviral construct that overexpressed FBW7 and then were cultured with 30 mmol/L of glucose for 48 hours.

### 2.2. Western Blot

Total proteins were extracted from renal mesangial cells using a total protein extraction kit (Roche, USA). The protein concentration was determined by means of a BCA Protein Assay kit (Bioworld Technology, USA). Western blotting was performed as described previously [14]. Immunoblot analysis was performed with primary antibodies as follows: mouse anti-LC3 antibodies (1:1000; Abcam, UK), mouse anti-FBW7 antibodies (1:1000; Abcam), rabbit anti-p-mTOR antibodies (1:1000; CST, USA), rabbit anti-mTOR antibodies (1:1000; CST), and anti-GADPH antibodies (1:8000; Bioworld Technology, USA). After washing with phosphate-buffered saline with Tween 20, blots were incubated with horseradish peroxidase- (HRP-) conjugated secondary antibodies for 1 hour. Proteins were detected with HRP chemiluminescence reagent (Millipore, USA). An automatic gel imaging system was utilized for image acquisition, and Quantity One software was applied to analyze the relative expression of proteins.

### 2.3. Immunofluorescence

SV40 MES 13 cells were grown in a confocal culture dish (Thermo, USA). When adherent cells constituted approximately 20% of the plate, cells were transfected with an mRFP-GFP-LC3 construct (HANBIO, China), according to the manufacturer's instructions. At 24 hours after transfection, GFP and RFP expressions were observed under a confocal laser scanning microscope (×40); mRFP was used to label and track LC3. Yellow spots represented autophagosomes (mRFP + GFP), and red spots were indicative of autolysosomes (mRFP).

### 2.4. Gene Overexpression

We utilized exogenous double-stranded RNA to mediate intracellular mRNA-specific degradation, thereby silencing expression of the target gene. This process yielded functional phenotype deletions. When adherent cells constituted approximately 30% of the plate, cells were transfected with a lentiviral construct carrying the FBW7 gene. At 8 to 12 hours after transfection, the culture fluid was changed. Green fluorescence was observed under a fluorescence microscope at 3 or 4 days after transfection. The best lentivirus titer obtained was 2 × l0^7^ TU/mL [multiplicity of infection (MOI), 20].

### 2.5. RNA Extraction and Reverse-Transcription PCR

Total RNA was extracted from renal mesangial cells in each group by means of an RNA extraction kit (Tiangen Biotech (Beijing), China). Isolated RNA was reverse-transcribed with a reverse-transcriptase kit (Toyobo, Japan). The reaction system and reaction conditions recommended by the manufacturer were utilized. Primer sequences were as follows: FBW7, forward, 5′-TAT CCG AAA CCT CGT CAC-3′, reverse, 5′- ACA TCA AAG TCC AGC ACC-3′; *β*-actin, forward, 5′-ACC TCT ATG CCA ACA CAG-3′, reverse, 5′-GGA CTC ATC GTA CTC CTG-3′.

### 2.6. Enzyme-Linked Immunosorbent Assay

Levels of Col-I, IL-1*β*, and caspase-1 were determined with enzyme-linked immunosorbent assay (ELISA) kits (Beijing Cheng Lin Biotechnology, China). The standard curve and regression equation were applied to calculate the sample density, according to the standard concentration and optical density (OD) values.

### 2.7. Statistical Analysis

Data were expressed as the mean ± standard deviation (SD) for at least 3 independent experiments. Differences among groups were analyzed with SPSS 17.0 statistical software by one-way ANOVA method.* P*<0.05 was regarded as statistically significant.

## 3. Results

### 3.1. High Glucose Decreased FBW7 in Renal Mesangial Cells

**Figure 1:**
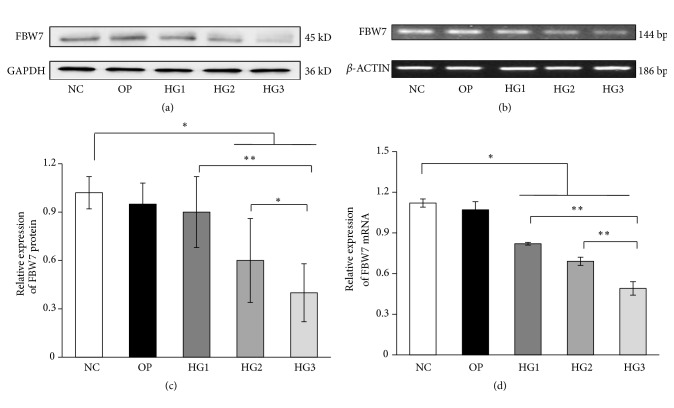
The expression of FBW7 protein and mRNA in renal mesangial cells induced by different concentrations of glucose for 48h. (a) FBW7 protein in renal mesangial cells induced by different concentrations of glucose and detected by western blot. (c) FBW7 mRNA detected by RT-PCR for different concentrations of glucose. (b, d) The corresponding relative gray value statistics graph of FBW7 level. NC group was treated with 5.6mmol/L glucose. OP group was treated with 5.6mmol/L glucose and 24.4mmol/L mannitol. HG1 group was treated with 10 mmol/L glucoses. HG2 group was treated with 20 mmol/L glucoses. HG3 group was treated with 30 mmol/L glucoses. *∗P* < 0.05 and *∗∗P* < 0.01.

FBW7 is an E3 ubiquitin ligase and a tumor suppressor; it played an important role in various cellular processes, including cell cycle progression, growth, differentiation, apoptosis, tumor metastasis, and tumor resistance. Compared with the NC group, FBW7 protein and mRNA decreased gradually with increasing glucose concentration for 48 h (*P*<0.05) (Figures 1(a)-1(d)). However, we found no difference between the OP and NC groups (*P*>0.05).

### 3.2. High Glucose Reduced Autophagy by Activating mTOR Signaling

**Figure 2:**
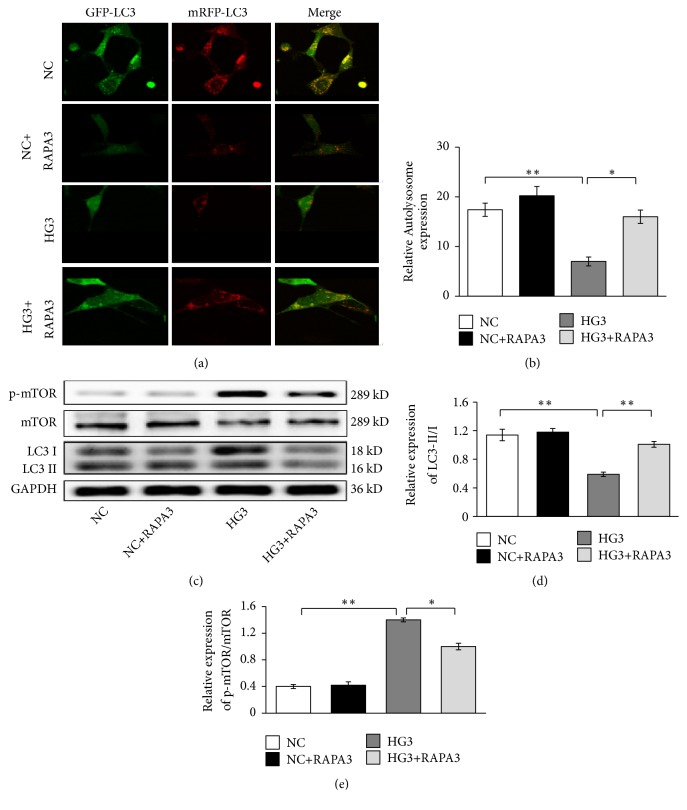
Effects of 30 mmol/L high glucose and rapamycin on mTOR/autophagy signaling in renal mesangial cells. (a) Autolysosome detected by confocal laser scanning microscope. (b) Quantitative analyses of number of autolysosomes. (c) mTOR, p-mTOR, and LC3-II/I detected by western blot. (d) The corresponding relative gray value statistics graph of LC3B. (e) The corresponding relative gray value statistics graph of p-mTOR/mTOR. *∗P* < 0.05 and *∗∗P* < 0.01.

The level of autophagy is reflected by the ratio of LC3-II/I and the number of autolysosomes. The expressions of mTOR, p-mTOR, LC3-II, and LC3-I were assessed by western blot. The number of autolysosomes was determined by confocal laser scanning microscopy, and the number of yellow blips represents the number of autolysosomes. Compared with the NC group, the relative expression of p-mTOR/mTOR increased in high-glucose groups for 48 h (*P*<0.05). On the contrary, the quantity of autolysosomes and the LC3-II/I ratio were significantly decreased in cells exposed to 30 mmol/L glucoses for 48 h (*P*<0.01) (Figures 2(a)-2(e)).

Rapamycin (RAPA) can combine with the FKBP-12 receptor to form a special complex that can bind the FRB domain of mTOR protein and specifically inhibit mTOR [15]. To investigate whether the mTOR protein is involved in the regulation of autophagy, we applied 200 nmol/L of RAPA as an inhibitor of mTOR. Compared with the HG3 group, the relative expression of p-mTOR/mTOR was suppressed obviously in the HG3-RAPA3 group (*P*<0.01) (Figures 2(c) and 2(e)); at the same time, the expression of LC3-II/I and autolysosome increased significantly (*P*<0.01) (Figures 2(a)-2(d)). We did not detect any difference between the NC group and the NC-RAPA3 group in these experiments (Figures 2(a)-2(e)).

### 3.3. RAPA Decreased Inflammatory and Fibrotic Cytokines by Inhibiting mTOR

**Figure 3:**
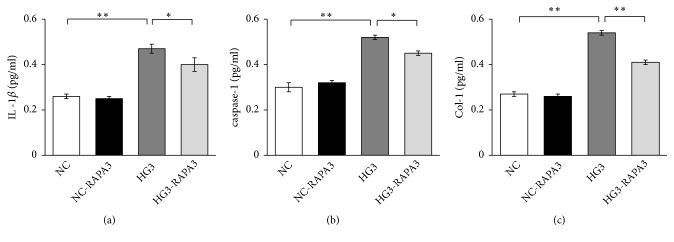
Effects of high glucose and rapamycin on the expression of inflammatory and fibrotic factors in renal mesangial cells. All factors were detected by ELIS. (a) IL-1*β*; (b) caspase-1; (c) Col-I. *∗P* < 0.05 and *∗∗P* < 0.01.

Inflammation and fibrosis take part in the progress of DN. Col-I is a fibrotic cytokine; IL-1*β* and caspase-1 are inflammatory cytokines. ELISA analysis was used to detected the expressions of Col-I, IL-1*β*, and caspase-1 in renal mesangial cells. And we found that they were higher in the high-glucose groups (HG3) than that in the NC group for 48 h (P<0.01), but they were decreased in HG3-RAPA3 group (*P*<0.01), and we did not detect any difference between the NC group and the NC-RAPA3 group in these experiments (Figures 3(a)-3(c)).

### 3.4. FBW7 Gene Overexpression Ameliorated Inflammation and Fibrosis by Activating Autophagy in Mesangial Cells

**Figure 4:**
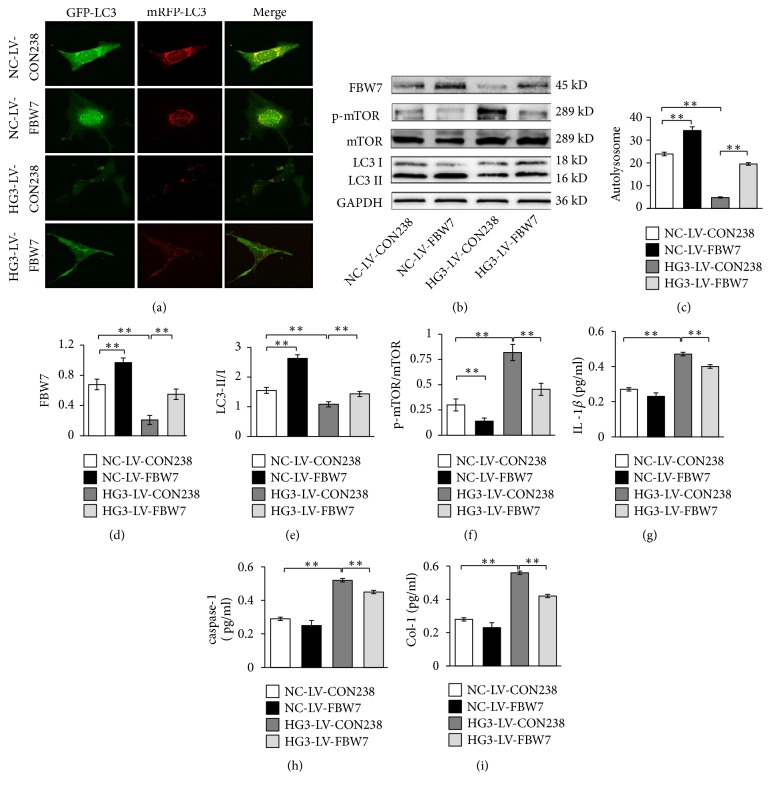
Effects of overexpression of FWB7 on mTOR/autophagy signal, inflammation, and fibrosis factors. (a) Autolysosome detected by confocal laser scanning microscope. (c) Quantitative analyses of number of autolysosomes. (b) FBW7, p-mTOR, mTOR, and LC3B detected by western blot. (d-f) The corresponding relative gray value statistics graph of the protein level. (g-i) The expression of inflammatory and fibrotic factors in renal mesangial cells detected by ELIS. (g) IL-1*β*; (h) caspase-1; (i) Col-I. *∗*P < 0.05 and *∗∗*P < 0.01.

FBW7 can be used to target the degradation of the mTOR protein; furthermore, it can promote the degradation of mTOR protein in breast cancer and renal cancer. To determine whether mTOR protein is regulated by FBW7 in mesangial cells, we subjected cells to FBW7 gene overexpression experiments. After FBW7 gene overexpression of mesangial cells was induced by 30mmol/L glucose for 48 h, FBW7 was overexpressed in HG3-LV-FBW7 groups (*P*<0.01). The relative expression of p-mTOR/mTOR was inhibited; furthermore, LC3-II/I and the quantity of autolysosomes increased in the HG3-LV-FBW7 group (*P*<0.01) (Figures 4(a)-4(f)). And the expressions of Col-I, IL-1*β*, and caspase-1 decreased significantly in HG3-LV-FBW7 group, compared with the HG3-LV-CON238 group (*P*<0.01) (Figures 4(g)-4(i)).

## 4. Discussion

Inflammation and fibrosis are important pathophysiologic processes in DN [16]. In DN, ECM accumulates in the renal glomerulus and renal tubule; this yields renal fibrosis and irreversible loss of renal tissue. Collagen-1 (Col-1) is the main component of ECM, which is closely related to cell growth, differentiation, proliferation, repair of tissue injury and inflammation, sclerosis, and fibrosis. Abnormal synthesis of Col-I can increase the production of reactive oxygen species (ROS) in DN and can increase the level of oxidative stress, resulting in progressive glomerular injury [17, 18]. IL-1*β* can also regulate the proliferation of mesangial cells, the production of ECM, and renal fibrosis. Caspase-1 catalyzes maturation and activation of the IL-1*β* precursor [19]. Hyperglycemia can yield inflammation and can induce renal injury by promoting IL-1*β* and caspase-1 expression in renal cells. Other authors determined that IL-1*β* and caspase-1 were increased in the kidney in a model of DN [20]. Restraining the activity of caspase-1 can inhibit inflammatory processes and delay the progression of DN [21]. Furthermore, caspase-1 precipitates release of inflammatory cytokines into the ECM, which concentrate and activate immune cells, thereby inducing an inflammatory cascade and promoting the development of DN [22]. Our results show that high glucose can upregulate the expressions of caspase-1, IL-1*β*, and Col-I. The results of this study demonstrate that inflammation and fibrosis are involved in the development of DN.

Autophagy is a process of self-swallowing in which cellular components are transported to lysosomes for degradation into the respective elementary materials. Autophagy also constitutes a mechanism of defense and stress induction against external stimuli and internal changes. In this role, autophagy functions in self-protection, maintaining cellular homeostasis and renewing organelles. When cells are invaded by bacteria or viruses, autophagy is carried out to degrade the pathogen as well as to prepare antigens to prime the innate and adaptive immune responses [23]. The process of autophagy can stabilize atherosclerotic plaques by preventing macrophage apoptosis and plaque necrosis [24]. Furthermore, autophagy can degrade cell components damaged by oxidative stress and can pare excessive collagen fibers [25]. By undergoing autophagy, cells can reduce insulin resistance and mitochondrial damage while also improving function of islet beta cells [26].

The studies suggested that a reduction in autophagy may be involved in the pathogenesis of DN, and protracted high-glucose stimulation can decrease the activity of autophagy in podocytes, leading to the development of DN [27]. Autophagy can alleviate inflammatory reactions in the kidney and can reduce the infiltration of inflammatory cells to delay the progression of renal interstitial fibrosis [28]. Autophagy also may protect kidneys from fibrosis by degrading intracellular collagen fibers [29]. The process of autophagy degrades excessive Col-I to relieve renal fibrosis [30].

The process of autophagy is inducible. In early-stage DN, autophagy can be induced to activate protective processes in response to short-term glucose stimulation. Autophagy is enhanced in mesangial cells and may be responsible for reduced deposition of extracellular matrix (ECM) and alleviated inflammation. Autophagy occurs rapidly: the autophagosome can be observed 8 minutes after stimulation and is degraded within 2 hours. These kinetics are conducive for the cell to adapt to external stimuli and affect rapid internal changes [31]. However, in disease, autophagy can be diminished. Kitada et al. found that the level of autophagy was decreased in an animal model of diabetes and in mesangial cells cultured under long-term high-glucose conditions [32]. Our results show that the quantity of autolysosomes and the LC3-II/I ratio decreased after induction with 30 mmol/L of glucose for 48 hours in renal mesangial cells; hence, high-glucose conditions will downregulate autophagy. These results are consistent with those of many other studies and suggest that protracted high-glucose stimulation inhibits autophagy [33].

The regulation of autophagy level is mainly achieved by regulating the mammalian target of rapamycin (mTOR), which is a Ser/Thr protein kinase with a molecular weight of 289 kDa. Phosphorylation of mTOR may occur, such as at Ser2448. Phosphorylated mTOR can facilitate cell proliferation and protein synthesis by activating downstream target molecules. A classic negative regulator of autophagy, mTOR can inhibit autophagy by interacting with Atg13 [34] or by inhibiting the activity of the autophagy-related protein 1 (ATGl)/ULKl complex [35]. Activation of the mTOR signaling pathway is an important event in the development of DN because activated mTOR can promote the release of cytokines and the transformation of renal tubular epithelial cells into fibroblasts [36]. Overexpression of mTOR facilitates disease progression. Inhibiting the expression and activity of mTOR can reduce insulin resistance and prevent glomerular hypertrophy, basement membrane thickening, and albuminuria [37]. Our results indicate that mTOR signal is involved in the regulation of autophagy in renal mesangial cells under high-glucose stimulation, and protracted exposure of cells to high glucose can cause upregulation of mTOR and p-mTOR expression.

Rapamycin as an inhibitor of mTOR has been shown to enhance autophagy activity by binding to FKBP-12 receptors to form a special complex in many studies. It is not clear whether rapamycin has the protective effect on DN. Some researchers had found that rapamycin can decrease intrarenal oxygen availability and alter glomerular permeability and slow the progress of DN [38]. But the mechanism was unclear. Based on renal inflammation and fibrosis, our study confirmed that rapamycin inhibited mTOR signal, increased the level of autophagy, and attenuated inflammation and fibrosis. Further molecular mechanisms and the molecules upstream of mTOR have not been well clarified.

FBW7 (also known as FBXW7, hCdc4, hAgo, and SEL10) is the substrate recognition component of the SCF ubiquitin ligase complex that connects ubiquitin with target proteins and is involved in cell growth, proliferation, differentiation, and apoptosis [39]. FBW7 comprises multiple protein-interaction domains that harbor WD40 repeats and D domains and also induces the direct binding of SCF to S-related protein kinase 1. Expression of the FBW7 gene is highly responsive but mutable to external stimuli [40]. Self-regulation of the FBW7 gene is often carried out at the levels of transcriptional (NF-*κ*B1) and posttranslational phosphorylation (ERK kinase and others) [41, 42], but the function is abnormal and then caused diseases. In our study, we found that the high glucose could decrease the expression of FBW7.

Other authors have shown that mTOR can be used as a substrate protein of FBW7 and can be modified by ubiquitination and degraded by proteasomes [9]. Our study confirmed that FBW7 plays a role similar to rapamycin to enhance the autophagy level by degradation of mTOR. We found that overexpression of FBW7 can significantly increase the expression of FBW7, autolysosomes, and LC3-II/I and decrease the relative expression of p-mTOR/mTOR, Col-I, IL-1*β*, and caspase-1. Our results indicate that decreased autophagy activity is related to inhibition of FBW7 expression in renal mesangial cells induced by high glucose. Therefore, FBW7 can regulate mTOR signal in mesangial cells induced by high glucose.

In conclusion, our study demonstrates that high glucose can downregulate the expression of FBW7, activate mTOR signal, and decrease the level of autophagy in renal mesangial cells. And FBW7 gene overexpression can increase autophagy by inhibiting mTOR signaling and ameliorate inflammation and fibrosis. Our findings suggest that FBW7 may be involved in the pathogenesis of DN by regulating the autophagy signaling pathway.

## Data Availability

(1) The data used to support the findings of this study are included within the manuscript. (2) The data used to support the findings of this study are included within the supplementary information file(s). (3) The data used to support the findings of this study were supplied by Yong Xu under license and so cannot be made freely available. Requests for access to these data should be made to Yong Xu [email: xywyll@aliyun.com]. (4) The data used to support the findings of this study are currently under embargo, while the research findings are commercialized. Requests for data [6/12 months] after publication of this article will be considered by the corresponding author. (5) The data used to support the findings of this study are available from the corresponding author upon request.
